# Next-generation-sequencing-based identification of familial hypercholesterolemia-related mutations in subjects with increased LDL–C levels in a latvian population

**DOI:** 10.1186/s12881-015-0230-x

**Published:** 2015-09-28

**Authors:** Ilze Radovica-Spalvina, Gustavs Latkovskis, Ivars Silamikelis, Davids Fridmanis, Ilze Elbere, Karlis Ventins, Guna Ozola, Andrejs Erglis, Janis Klovins

**Affiliations:** Latvian Biomedical Research and Study Center, Ratsupites Street 1, Riga, LV-1067 Latvia; Latvian Center of Cardiology, Pauls Stradins Clinical University Hospital, Pilsonu Street 13, Riga, LV-1002 Latvia; Faculty of Medicine, University of Latvia, Raina Blvd. 19, Riga, LV-1586 Latvia; Research Institute of Cardiology, University of Latvia, Pilsonu Street 13, Riga, LV-1002 Latvia; Vidzemes Hospital, Jumaras Street 195, Valmiera, LV-4201 Latvia

**Keywords:** APOB, Diagnostic tools, Genetics, LDL, LDLR, LDLRAP1, Next-generation sequencing, PCSK9

## Abstract

**Background:**

Familial hypercholesterolemia (FH) is one of the commonest monogenic disorders, predominantly inherited as an autosomal dominant trait. When untreated, it results in early coronary heart disease. The vast majority of FH remains undiagnosed in Latvia. The identification and early treatment of affected individuals remain a challenge worldwide. Most cases of FH are caused by mutations in one of four genes, *APOB*, *LDLR*, *PCSK9*, or *LDLRAP1*. The spectrum of disease-causing variants is very diverse and the variation detection panels usually used in its diagnosis cover only a minority of the disease-causing gene variants. However, DNA-based tests may provide an FH diagnosis for FH patients with no physical symptoms and with no known family history of the disease. Here, we evaluate the use of targeted next-generation sequencing (NGS) to identify cases of FH in a cohort of patients with coronary artery disease (CAD) and individuals with abnormal low-density lipoprotein–cholesterol (LDL–C) levels.

**Methods:**

We used targeted amplification of the coding regions of *LDLR*, *APOB*, *PCSK9*, and *LDLRAP1*, followed by NGS, in 42 CAD patients (LDL–C, 4.1–7.2 mmol/L) and 50 individuals from a population-based cohort (LDL–C, 5.1–9.7 mmol/L).

**Results:**

In total, 22 synonymous and 31 nonsynonymous variants, eight variants in close proximity (10 bp) to intron–exon boundaries, and 50 other variants were found. We identified four pathogenic mutations (p.(Arg3527Gln) in APOB, and p.(Gly20Arg), p.(Arg350*), and c.1706–10G > A in LDLR) in seven patients (7.6 %). Three possible pathogenic variants were also found in four patients.

**Conclusion:**

NGS-based methods can be used to detect FH in high-risk individuals when they do not meet the defined clinical criteria.

**Electronic supplementary material:**

The online version of this article (doi:10.1186/s12881-015-0230-x) contains supplementary material, which is available to authorized users.

## Background

Familial hypercholesterolemia (FH) was first described in 1920 [1] and is possibly the most common single-gene disorder. FH is characterized by elevated low-density lipoprotein–cholesterol (LDL–C) levels, tendon xanthoma, and arcus corneae before the age of 45 years. In the majority of cases, FH is inherited as an autosomal dominant trait, with a penetrance of almost 100 % [2]. Based on the Copenhagen General Population Study, the prevalence of heterozygous FH is about one in 200 individuals. However, the prevalence varies from one in 200 to one in 500 individuals, depending on the population [3]. The homozygous form of FH is much rarer, occurring in only one in 1,000,000 individuals [4–6].

The accumulation of lipids and lipoproteins in the plasma is the first key process in the development of atherosclerotic lesions [7]. Atherosclerosis, its resultant coronary artery disease (CAD), and other vascular diseases are the commonest causes of deaths worldwide [8], and the early diagnosis of FH patients before the development of severe complications is crucial to the commencement of appropriate treatment with lipid-lowering agents.

The diagnosis of FH is often based on physical signs (tendon xanthoma or arcus corneae before 45 years of age), laboratory findings (plasma LDL–C levels), and the patient’s history of elevated LDL–C and cardiovascular disease. There are at least three approaches to the diagnosis of FH based on these signs [9]. However, the absence of physical symptoms is not sufficient to reject a diagnosis, because about 50 % of heterozygous FH patients do not display physical signs [10] and there is often no evidence of premature CAD in heterozygous FH patients [4, 11]. Moreover, in some monogenic hypercholesterolemias, lipid plasma levels are similar to the normal ranges [5]. Therefore, DNA-based tests are required to confirm a diagnosis of FH.

In majority of FH patients have a defective low-density lipoprotein receptor (LDLR). This results in the insufficient uptake of low-density lipoprotein (LDL) particles by cells and the accumulation of LDL–C in the plasma [12, 13]. Extremely elevated plasma LDL–C levels greatly increase the risk (by more than 50 %) of premature CAD before the age of 55 years [11–14]. Hundreds of *LDLR* mutations have been reported and many of them cause FH (http://www.ucl.ac.uk/ldlr/Current/, http://www.hgmd.org/). A phenotypically similar condition, familial defective apolipoprotein B (FDB), is caused by defects in the apolipoprotein B gene (*APOB*) [12, 15], and especially by mutations in the LDLR-binding domain of APOB [14, 16]. These mutations prevent the recognition of the APOB protein by LDLR, and therefore LDL particles bound to defective APOB cannot be taken up by cells. Various mutations in the APOB protein are related to FDB, most commonly p.(Arg3527Gln) [15, 17, 18]. Gain-of-function mutations in proprotein convertase subtilisin/kexin type 9 (PCSK9) have been suggested to increase the degradation of LDLR, reducing the number of LDLR molecules on the cell surface. This further reduces the uptake of LDL particles by cells, leading to yet another type of monogenic hypercholesterolemia [13, 19–21]. There is also evidence that functional mutations in low-density lipoprotein adapter protein 1 (LDLRAP1) cause a recessive form of FH [22, 23]. The plasma LDL–C levels in these patients tend to be intermediate compared with those in patients with heterozygous or homozygous familial LDLR deficiency [24]. This adaptor protein is crucial for the normal assembly of LDLR-rich coated pits on the cell surface and therefore for normal LDL particle uptake into the cell by endocytosis [23, 25].

The proportion of patients identified with FH and under treatment remains very low, and most undiagnosed and untreated patients are young [13]. Recent findings also suggest that the prevalence of FH is higher than previously thought [3]. In this context, DNA-based evidence of functional mutations in the *LDLR*, *APOB*, *PCSK9*, and *LDLRAP1* genes is pivotal to making a correct diagnosis [26] because the actual prevalence of FH may be underestimated with the current diagnostic criteria for FH [3].

We conducted this study to assess the spectrum of FH-related mutations in a group of individuals that was selected mainly based on their elevated LDL–C levels, thus representing a typical population, including many individuals with undiagnosed FH. Another aim of the study was to estimate the feasibility of targeted next-generation sequencing (NGS)-based testing for FH.

## Methods

### Subjects

We conducted this research using DNA samples from the Genome Database of the Latvian Population (LGDB), a government-funded biobank (described briefly in [27]) that included 25,292 participants in August 2014 (1.3 % of the Latvian population). In the LGDB, 4335 samples included data on total cholesterol (TC), high-density lipoprotein–cholesterol (HDL–C), LDL-C, and triglyceride (TG) levels. Two groups of patients were included in the study. We selected 50 unrelated individuals from the extreme end of the LDL–C spectrum and designated them the “population group” (POP). In total, 292 individuals had LDL–C levels > 4.9 mmol/L, which is the cut-off value for LDL-C based on World Health Organization criteria [11]. In this dataset, 50 samples from the upper end of the distribution, representing a range of LDL–C from 5.1 to 9.7 mmol/L, were chosen. The second group of 42 patients was recruited to the LGDB from the Latvian Center of Cardiology, the “CAD” group, during the period to March 2013, specifically for this study. The inclusion criteria for this group was a history of coronary heart disease (previous myocardial infarction or angina) and/or evidence of coronary atherosclerosis, established with invasive coronary angiography, and a TC level > 7 mmol/l. All subjects who gave their consent to participate in the study were included without further selection. All individuals from the CAD group underwent lipid-lowering therapy and highest known LDL-C value independently of pre- or post- treatment was used for this study. Written informed consent was obtained from all the participants. The study protocol was approved by the Central Medical Ethics Committee of Latvia (protocols nr. 2007 A-7 and 01–29.1/25). Sixteen (17 %) samples were also included in the previous pilot study, investigated with the same sequencing panel [28].

### Next-generation sequencing (NGS)

We sequenced all the exons (including the exon–intron boundaries), and 5′ and 3′ untranslated regions of four FH-related genes (*LDLR*, *APOB*, *PCSK9*, and *LDLRAP1*), with the Life Technologies Ion Torrent™ PGM platform, which is based on the semiconductor sequencing technology [29]. For target enrichment, we developed an AmpliSeq™ assay, using the Ion AmpliSeq™ Designer tool to custom design the assay primers. The design was based on the human (Hg19) reference genome and pipeline v1.2 to generate 200-bp amplicons. In total, 192 primer pairs were designed and mixed in two pools. The primer pools were manufactured by Life Technologies and the theoretical coverage of the regions of interest was 92.1 %. An Invitrogen Qubit 2.0 fluorometer was used to normalize the genomic DNA concentrations to 5 ng/μl. A standard barcoded AmpliSeq™ library preparation protocol, with 10 ng of input DNA (release: 10 September 2012, publication part nr. MAN0006735), was used to prepare the DNA libraries. The final libraries were quantified and their quality checked on an Agilent 2100 Bioanalyzer using High Sensitivity DNA chips (Agilent Technologies). The libraries were diluted to a concentration of approximately 13 pM and all of the 16 libraries were pooled to ensure the efficient use of the Ion chip space. The Ion OneTouch™ 200 Template Kit v2 DL (release: 12 September 2012, publication nr. MAN0006957) was used for template preparation and emulsion PCR. The Ion PGM™ 200 Sequencing Kit (release: 26 September 2012, Rev. F, publication nr. 4474246) and the standard sequencing protocol for the Ion 314™ chip were used for the sequencing procedure. The predicted average coverage was 30x (according to the standard AmpliSeq protocol).

### Variant detection and analysis

The raw data acquired were aligned to the human reference genome (Hg19) using the Torrent suite 4.0.2 alignment plugin v4.0-r77189. The variant-calling plugin (variantCaller v4.0-r76860) was used to call and annotate the detected variations within the sequenced samples. The Standard–Germ Line–PGM–Low Stringency settings were chosen for the variant-calling plugin, in which the minimal coverage for calling single-nucleotide polymorphisms (SNPs) was 6x. All variants called were visually inspected using the Integrative Genomics Viewer (IGV) v2.3 software (http://www.broadinstitute.org/software/igv/download) [30] to filter out possible PCR errors or false-positive variants caused by imperfections in the variant-calling plugin algorithms or by the platform specificity [31].

Variants with minor allele frequencies above 1 %, according to the 1000 Genomes phase 1 genotype data from 1094 worldwide individuals (http://browser.1000genomes.org), were considered nonpathogenic. Similarly, all deep intronic variants (more than 10 bp away from exon–intron boundaries), other noncoding variants, and synonymous variants were excluded from further analysis. The remaining variants were analyzed by comparing them with the Human Gene Mutation Database (HGMD; http://www.hgmd.org/), the Familial Hypercholesterolemia Variant Database (FHVD; http://www.ucl.ac.uk/ugi/fh), and scientific publications, for annotation purposes. Annotation *in silico* was also performed using tools that predict the functional effects of human SNPs: PolyPhen-2 v2.2.2r398 [32], SIFT [33], and Mutation Taster [34]. The variants described as causing FH in the FHVD, supported by evidence in the literature, were considered pathogenic (Category 1). Nonsynonymous variants with unknown or unclear evidence of pathogenicity, which were identified as damaging by all the prediction tools, were considered possibly pathogenic (Category 2). All the remaining variants were classified as variants of uncertain clinical significance (Category 3). All the Category 1 and Category 2 variants were directly sequenced for validation.

## Results

For the purposes of this research, we selected 50 patients who represented the general population, with markedly elevated LDL–C levels (among these, only one individual had a prior history of CAD), the POP group, and 42 patients with elevated LDL–C levels and established CAD, the CAD group. The clinical characteristics of our cohort are summarized in Table 1.

**Table 1:** Clinical characteristics of our study cohort

Variable	CAD	POP	*P*-value	Total
n	42	50		92
Age, years ± SD (min-max)	60.0 ± 12.1 (41–89)	58.6 ± 9.7 (23–78)	0.0017	62.0 ± 11.4 (23–89)
Female gender, %	35.7	68.0	0.0023	52.2
CAD, %	100	2.0	<0.0001	46,7
MI, %	54.8	0.0	<0.0001	25,0
TC level, mmol/L ± SD (min-max)	7.9 ± 0.7 (6.7-10.1)	9.3 ± 1.6 (7.0-14.0)	<0.0001	8.7 ± 1.5 (6.7-14.0)
LDL-C level, mmol/L ± SD (min-max)	5.6 ± 0.7 (4.1-7.2)	6.6 ± 1.1 (5.1-9.7)	<0.0001	6.2 ± 1.0 (4.1-9.7)
LDL-C > 8.5 mmol/L,n	0	5	0.0238	5
LDL-C 6.5-8.4 mmol/L,n	7	18	0.0343	25
LDL-C 5.0-6.4 mmol/L,n	30	27	0.0855	57
LDL-C 4.0-4.9 mmol/L,n	5	0	0.0235	5
BMI, kg/m^2^ ± SD (min-max)	28.8 ± 4.6 (16.9-41.5)	28.0 ± 5.8 (16.9-42.6)	0.4647	28.3 ± 5.3 (16.9-42.6)

*CAD* – coronary artery disease; *MI* – myocardial infarction; *TC* – total cholesterol; *LDL-C* – low density lipoprotein cholesterol; *BMI* – body mass index; For age, TC, LDL-C and BMI mean values, ± standard deviations (SD) are given as well as minimal and maximal values in brackets

After sequencing errors caused by PCR mistakes, variants called due to inadequate variant-calling algorithms near homopolymers, and strand-specific errors specific to the Ion Torrent platform [31] were removed, 114 different variants were detected in the total study group. For three variants, the alternative allele frequencies were 100 %. These were assumed to be inappropriately annotated in the reference genome and were not included in the list of variants [35]. Similarly, synonymous coding variants (*n* = 22) were not included in the list of variants. In total, 31 nonsynonymous variants, eight variants in close proximity (10 bp) to intron–exon boundaries, and 50 other variants were identified. Information on all (*n* = 89) the identified variants is given in Additional file 1: Table S1. Four FH-causing variants (Category 1), three possibly pathogenic variants (Category 2), and eight variants of uncertain significance (Category 3) were identified (shown in Table 2). The target coverage achieved in the regions of interest was 90.7 % and the mean read depth was 117×.

**Table 2:** Variants of definite and possible importance found in suspected FH patient cohort of Latvian population

CAT	Gene	rs code	AAF	FRQ	Variants	Description with references
1	*APOB*	rs5742904	T = 0,016	T = 0,001	p.(Arg3527Gln)	Hypercholesterolemia [18, 40, 41, 44, 49, 50, 52], associated with increased LDL-C [51]
1	*LDLR*	rs147509697	A = 0,011	A = 0,001	p.(Gly20Arg)	Hypercholesterolaemia, possibly damaging [38], found in FH patient [39–43], used in LIPOCHIP – FH diagnosis panel [44]
1	*LDLR*		T = 0,005		p.(Arg350*)	Hypercholesterolaemia, truncated peptide [13, 36, 37, 50]
1	*LDLR*	rs17248882	A = 0,005	A = 0.002	c.1706-10G > A	Hypercholesterolaemia? [45, 47], 3’splice acceptor mutation in intron 11 [46, 48], computed estimation – outside splicing regulatory regions [38], found in seven FH patients [40, 49]
2	*APOB*	rs201990496	C = 0,005	A = 0.000	p.(Ser3915Cys)	No information found
2	*APOB*	rs151009667	T = 0,011	T = 0.002	p.(Arg1689His)	Hypertriglyceridaemia?[53]
2	*APOB*		G = 0,005		p.(Tyr144His)	No information found
3	*APOB*	rs72654423	C = 0,005	C = 0.003	p.(Ile4314Val)	Found in individuals with high TG levels [63]
3	*APOB*	rs61743502	G = 0,005	G = 0.003	p.(Val4265Ala)	No information found
3	*APOB*	rs1801696	T = 0,016	T = 0.002	p.(Glu2566Lys)	Hypertriglyceridaemia?[53]
3	*APOB*	rs72653092	T = 0,011	T = 0.001	p.(Ser2429Thr)	Hypertriglyceridaemia?[53]
3	*APOB*		T = 0,005		p.(Val2095Glu)	No information found
3	*APOB*		A = 0,005		p.(Met755Leu)	No information found
3	*APOB*	rs146152405	A = 0,005	T = 0.001	p.(Arg214Leu)	No information found
3	*LDLR*		G = 0,005		c.2141-9 T > G	No information found

*CAT* – our designated category of variant; *AAF* – alternative allele frequency in our cohort; *FRQ* – frequency of general population (http://www.ncbi.nlm.nih.gov/); Variant – amino acid exchange (amino acid numbering according to Human Genome Variation Society [64])

Four new variants were identified (not reported in any variant database): one missense change p.(Tyr144His) (APOB) from Category 2, and two missense changes p.(Val2095Glu) and p.(Met755Leu) (both in APOB) and one variant close to an *LDLR* splice site (c.2141–9 T > G) from Category 3. Table 3 shows the individual patients with Category 1, 2, and 3 variants.

**Table 3:** Individual patients with Category 1, 2 and 3 variants

Patient	CAT	Group	Age	Sex	LDL-C level, mmol/L	Gene ID	AA	PolyPhen-2	SIFT	Mutation taster
4	1	POP	49	M	5,92	*LDLR*	c.1706-10G > A	-	-	N
16	1	POP	62	M	8,15	*APOB*	p.(Arg3527Gln)	D	D/T	D
	3					*APOB*	p.(Glu2566Lys)	B/D	D/T	N
33	1	CAD	82	M	4,80	*LDLR*	p.(Gly20Arg)	B	D/T	N
52	1	CAD	81	M	7,16	*LDLR*	p.(Gly20Arg)	B	D/T	N
62	1	POP	23	F	6,23	*LDLR*	p.(Arg350*)	-	D	D
73	1	POP	65	F	7,56	*APOB*	p.(Arg3527Gln)	D	D/T	D
	3					*APOB*	p.(Glu2566Lys)	B/D	D/T	N
	3					*APOB*	p.(Val2095Glu)	B	D	N
85	1	POP	57	F	6,65	*APOB*	p.(Arg3527Gln)	D	D/T	D
	3					*APOB*	p.(Glu2566Lys)	B/D	D/T	N
14	2	CAD	57	M	6,60	*APOB*	p.(Arg1689His)	D	D	D
25	2	CAD	58	F	5,90	*APOB*	p.(Tyr144His)	P	D	D
32	2	CAD	66	M	5,28	*APOB*	p.(Ser3915Cys)	P/D	D	D
123	2	POP	48	M	5,89	*APOB*	p.(Arg1689His)	D	D	D
11	3	CAD	73	M	5,10	*APOB*	p.(Arg214Leu)	P	D/T	N
12	3	CAD	62	M	6,06	*APOB*	p.(Ser2429Thr)	B	T	N
30	3	CAD	59	F	5,76	*APOB*	p.(Ser2429Thr)	B	T	N
42	3	POP	65	M	5,16	*APOB*	p.(Val4265Ala)	B	D	N
56	3	CAD	61	M	5,17	*APOB*	p.(Met755Leu)	B	T	N
90	3	POP	61	F	6,51	*APOB*	p.(Ile4314Val)	B	D/T	N
118	3	POP	58	F	5,95	*LDLR*	c.2141-9 T > G	-	-	D

*CAT* – ours designated category of variant; *M* – male; *F* – female; *LDL-C* – low density lipoprotein cholesterol; *AA* – amino acid exchange (amino acid numbering according to Human Genome Variation Society [64]); *D* – probably damaging/damaging; *P* – possibly damaging; *B* – benign, *T* – tolerated; *N* – polymorphism

FH-causing mutations were identified in seven patients (7.6 %) and possibly pathogenic variants were found in another four patients. No significant difference between the CAD and POP groups was identified in the number of variants from Categories 1, 2, or 3. All Category 1 and Category 2 variants were verified with direct sequencing.

## Discussion

The heterogeneity of FH-causing variants supports the sequence-based detection of the disease as the primary methodology, and the recent advances in NGS-based techniques has made them more accessible for use in diagnostics. However, it is unclear how applicable this approach is to the identification of individuals with FH in primary care. In this study, we used our recently developed AmpliSeq-based assay [28] to test the ability of this approach to identify FH variants in high-risk groups from general and disease-specific populations, primarily selected on the basis of their available clinical and biochemical data. Therefore, the sole criterion for inclusion in the POP group was elevated LDL-C levels, whereas the CAD group was selected based on clinical or angiographic evidence of CAD and high TC levels (>7 mmol/l). The two groups showed significant differences in their LDL–C levels and the presence of CAD or myocardial infarction. However, the two groups did not differ in the number of variants identified (regardless of their proposed functions). We found four pathogenic FH-causing mutations in 7.6 % of the total group of subjects tested. The LDLR-protein-terminating mutation p.Arg350* was found in one subject. This variant has previously been reported as an FH-causing variant in 13 unrelated FH patients [13, 36, 37]. It encodes a truncated version of the protein, a nonfunctional LDLR, and therefore reduces the uptake of LDL particles in the liver and other tissues. p.(Gly20Arg) in LDLR was found in two patients and has previously been identified in FH patients in many studies [38–43]. Although it was predicted to be a benign variant by PolyPhen-2, LDLR p.(Gly20Arg) is already used in the LIPOchip®–FH diagnostic panel [44] and is a confirmed FH-causing variant. Similarly, c.1706 –10G > A in LDLR was identified by us in one patient, has been previously and repeatedly detected in FH patients [40, 45–49]; it is also used in an FH diagnostic panel [49]. Another variant, p.(Arg3527Gln) in APOB, was found in three subjects, and is the most common FH-causing mutation, supported by much epidemiological and functional evidence [18, 41, 50–52]. Interestingly, another APOB variant, p.(Glu2566Lys), was found in all three subjects, suggesting strong linkage disequilibrium between these variants.

Another three rare *APOB* mutations were suggested to be potentially damaging, in terms of the protein function, by all the prediction tools used in this study. One of these, p.(Arg1689His), has been described in the HGMD database as possibly causing hypertriglyceridemia. However, this variant has also been reported in control subjects [53], suggesting that it only moderately influences the FH phenotype. The other two rare variants of *APOB*, which have not been described previously, were found in two (2.2 %) subjects. In addition to the variants described above, we found seven rare (<1 % according to the 1000 Genomes phase 1 genotype data from 1094 individuals worldwide; http://browser.1000genomes.org) nonsynonymous variants of unknown clinical significance (Category 3). All nonsynonymous variants in Category 3 were located in the *APOB* gene. Apart from p.(Glu2566Lys) which is in LD with the pathogenic mutation p.(Arg3527Gln), only p.(Ser2429Thr) has been reported in relation to hypertriglyceridemia [53]. Three variants of *APOB*, p.(Val4265Ala), p.(Arg214Leu), and p.(Ile4314Val), are reported in the SNP databases, whereas two other variants of APOB, p.(Val2095Glu) and p.(Met755Leu), are new and have not been reported previously. The regions defined by amino acids 3174–3184 and 4181–4540 of APOB are important for the correct folding of the protein [54], suggesting that variants p.(Val4265Ala) and p.(Ile4314Val) detected in our study are very probably related to the FH phenotype. However, variants in other exons of the *APOB* gene, apart from its functional domains, are also known to be potentially pathogenic [55], so p.(Arg214Leu), p.(Met755Leu), p.(Val2095Glu), and p.(Ser2429Thr) may also be pathogenic. We also found one new variant located close to the *LDLR* donor splice site of exon 15 (c.2141–9 T > G). Category 3 variants were identified in 11 patients, representing 12.0 % of the variants from all subjects (Table 3). It should be noted that to assess the possible functions of the Category 2 and 3 variants, additional functional and segregation studies will be required to determine the pathogenicity of these variants. However, for variants with low penetrance, which is attributed to the APOB variants, this can be a difficult task and the challenge of interpreting rare variants identified with NGS is the main obstacle to the introduction of this technique to clinical diagnostics. In addition to rare variants, we also detected a number of common polymorphisms, including nonsynonymous variants that have been associated with elevated LDL–C, atherosclerosis, and other conditions (summarized in Additional file 1: Table S1). The extent to which the presence of these common variants influences the outcomes of patients with rare low-penetrance variants is unclear.

One of our aims in this project was to compare two groups that may be representative of populations of undiagnosed FH patients: the POP group, representing the general population, but with markedly elevated LDL–C levels, and the CAD group, representing coronary atherosclerosis patients, the majority of whom are on lipid-lowering therapy. Therefore, we did not attempt to control for equal LDL–C levels between the two groups. Our study group does not represent a typical FH cohort because no data were collected on their clinical phenotypes, including their tendon xanthomata (in the POP group), and no such phenotypes were observed (in the CAD group). Although some family histories were collected, they did not provide sufficient details to apply the commonly used FH criteria to define the disease. Another relevant consideration was the advanced age of most of the subjects included in the study, which may have caused variants with strong effects to be underrepresented. Thus, the pathogenic LDLR mutation p.(Arg350*), resulting in a nonfunctional protein, was identified in a 23-year-old patient, whereas the *APOB* variants known for their moderate effects were found in three patients aged 57–65 years. It is also well known that combinations of risk alleles of several small effect SNPs, can increase LDL–C levels [56–58]. Therefore, it cannot be excluded that LDL–C levels, the main selection criterion, were influenced by these SNPs in our cohort. Even more interesting is how these SNPs modify the LDL–C levels in patients with a monogenic background. However, much larger cohorts will be required to study the role of SNPs with sufficient statistical power. The main difference between the two groups was the presence of CAD in one of them. An increased prevalence of FH-related variants could be expected in the CAD group. However, we observed no significant differences in the numbers of subjects with Category 1, 2, or 3 variants between the groups (*p* = 0.684), although there was a clear tendency for these groups to differ in the number of carriers of specifically Category 1 variants. Even more interesting is the observation that an excess of carriers of Category 1 variants was found in the POP group (*n* = 5, 10.0 %), rather than in the CAD group (*n* = 2, 4.8 %) as one would expect. This suggests that the FH-related variant rate is higher in populations with higher LDL–C levels, irrespective of the presence of CAD. It is very likely that the rate of FH diagnosis would be even higher in a population selected with an inclusion criterion of a higher LDL–C cut-off. This is consistent with suggestions to increase the total cholesterol cut-offs to improve the diagnostic rate for FH [59]. However, such conclusions must be validated in larger cohorts because the majority of similar studies, including ours, acquired their data from very small cohorts and therefore lacked power.

The FH-related mutation detection rate of 7.6 % for definite FH is slightly higher than but similar to that in a recently published study in which FH-causing variants were identified in 2.1 % of unrelated high-risk individuals [60]. Studies in which NGS was used to detect variants in FH patient populations with defined clinical criteria reported higher detection rates, ranging from 67 % in clinically defined FH patients to 26 % in possible FH patients [61]. It is clear that studies with stronger inclusion criteria, based on clinical signs of FH or the presence of a family history, show much higher detection rates. The recent study by Maglio and colleagues [62] showed a detection rate of approximately 60 % in a study group in which all the participants had a family history of CAD, and 74 % of whom were classified with definite or probable FH, and 16 % displayed xanthomas.

A typical drawback of amplicon-based sequencing methods is the incomplete coverage arising from the limits of multiplexing. With our approach, 92.1 % of regions of interest were covered theoretically, whereas 90.7 % were obtained in practice because some amplicons did not produce PCR products. However, the depth of reads for all the successfully sequenced amplicons was sufficient to cover the expected regions completely. This can be considered close to optimal because all the known functional domains were covered and the regions omitted were mainly untranslated parts of the genes.

## Conclusions

In conclusion, we have shown that NGS-based methods can be used to detect FH in high-risk individuals and delivered a definitive diagnosis of FH in 7.6 % of all subjects in a population who did not meet the defined clinical criteria for FH. In this study, we also discussed the possible roles of low-penetrance variants in the etiology of high LDL–C and the problems associated with the interpretation of NGS results. It is clear that additional cascade testing in family members must be performed in subjects with uncertain variants. Implementation of the NGS-based approach may increase the percentage of known cases of FH, a currently underdiagnosed disease, and even more importantly, individuals with milder forms of FH and elevated LDL–C.

## Additional file

Additional file 1: Table S1. All variants found in study group. (DOC 611 kb)

## Abbreviations

*APOB*: Apolipoprotein B gene
APOB: Apolipoprotein B
CAD: Coronary artery disease
FDB: Familial defective apolipoprotein B
FH: Familial hypercholesterolemia
FHVD: The Familial Hypercholesterolemia Variant Database
HDL–C: High-density lipoprotein–cholesterol
HGMD: The Human Gene Mutation Database
LDL–C: Low-density lipoprotein–cholesterol
LDLR: Low-density lipoprotein receptor
LDLRAP1: Low-density lipoprotein receptor adapter protein 1
LGDB: The Genome Database of the Latvian Population
NGS: Next-generation sequencing
PCSK9: Proprotein convertase subtilisin/kexin type 9
SNP: Single-nucleotide polymorphism
TC: Total cholesterol
