# Supplementary material for: Next-generation-sequencing-based identification of familial hypercholesterolemia-related mutations in subjects with increased LDL–C levels in a latvian population
Source: BMC Med Genet. 2015 Sep 28;16:86. doi: 10.1186/s12881-015-0230-x (PMC4587402; doi:10.1186/s12881-015-0230-x)
Supplement: Additional file 1: Table S1. — All variants found in study group. (DOC 611 kb) [file 12881_2015_230_MOESM1_ESM.doc]

Supplementary Table 1. All variants found in study group.

| **CAT** | **Gene** | **rs code** | **AAF** | **FRQ** | **HET** | **HOM** | **Variant** | **Description with references** |
| --- | --- | --- | --- | --- | --- | --- | --- | --- |
| 1 | *APOB* | rs5742904 | T=0,016 | T=0,001 | 3 | 0 | p.(Arg3527Gln) | Hypercholesterolemia [1-7], associated with increased LDL-C [8] |
| 1 | *LDLR* | rs147509697 | A=0,011 | A=0,001 | 2 | 0 | p.(Gly20Arg) | Hypercholesterolaemia, possibly damaging [9], found in FH patient [10, 7, 1, 11, 12], used in LIPOCHIP – FH diagnosis panel [5] |
| 1 | *LDLR* |  | T=0,005 |  | 1 | 0 | p.(Arg350*) | Hypercholesterolaemia, truncated peptide [3, 13-15] |
| 1 | *LDLR* | rs17248882 | A=0,005 | A=0.002 | 1 | 0 | c.1706-10G>A | Hypercholesterolaemia? [16, 17], 3'splice acceptor mutation in intron 11 [18, 19], computed estimation – outside splicing regulatory regions [9], found in seven FH patients [7, 6] |
| 2 | *APOB* | rs201990496 | C=0,005 | A=0.000 | 1 | 0 | p.(Ser3915Cys) | No information found |
| 2 | *APOB* | rs151009667 | T=0,011 | T=0.002 | 2 | 0 | p.(Arg1689His) | Hypertriglyceridaemia?[20] |
| 2 | *APOB* |  | G=0,005 |  | 1 | 0 | p.(Tyr144His) | No information found |
| 3 | *APOB* | rs72654423 | C=0,005 | C=0.003 | 1 | 0 | p.(Ile4314Val) | Found in individuals with high TG levels [21] |
| 3 | *APOB* | rs61743502 | G=0,005 | G=0.003 | 1 | 0 | p.(Val4265Ala) | No information found |
| 3 | *APOB* | rs1801696 | T=0,016 | T=0.002 | 3 | 0 | p.(Glu2566Lys) | Hypertriglyceridaemia?[20] |
| 3 | *APOB* | rs72653092 | T=0,011 | T=0.001 | 2 | 0 | p.(Ser2429Thr) | Hypertriglyceridaemia?[20] |
| 3 | *APOB* |  | T=0,005 |  | 1 | 0 | p.(Val2095Glu) | No information found |
| 3 | *APOB* |  | A=0,005 |  | 1 | 0 | p.(Met755Leu) | No information found |
| 3 | *APOB* | rs146152405 | A=0,005 | T=0.001 | 1 | 0 | p.(Arg214Leu) | No information found |
| 3 | *LDLR* |  | G=0,005 |  | 1 | 0 | c.2141-9T>G | No information found |
| 4 | *APOB* | rs142151703 | A=0,005 | A=0.002 | 1 | 0 | c.*179G>A | No information but far away in 3' end |
| 4 | *APOB* |  | G=0,005 |  | 1 | 0 | c.*15A>G | No information found |
| 4 | *APOB* | rs1801695 | T=0,016 | T=0.016 | 3 | 0 | p.(Ala4481Thr) | Associated with HDL cholesterol [22], associated with risk for dementia [23], found in individuals with low TG levels [21] |
| 4 | *APOB* | rs1042034 | C=0,206 | C=0.338 | 30 | 4 | p.(Ser4338Asn) | Predicted as benign [24], interaction with other SNPs is associated with dietary cholesterol absorption [25], associated with TG and HDL cholesterol [26], common variant |
| 4 | *APOB* | rs1801702 | G=0,016 | G=0.062 | 3 | 0 | p.(Arg4270Thr) | Associated with TC and LDL-C [27], found in individuals with low TG levels [21] |
| 4 | *APOB* | rs1042031 | T=0,196 | T=0.153 | 28 | 4 | p.(Glu4181Lys) | Predicted as benign [24], associated with decreased LDL-C [28, 8], associated with calcific aortic valve stenosis [29], associated with HDL cholesterol [27], influences BMI [30], common variant, |
| **Supplementary Table 1 continuation** | | | | | | | | |
| 4 | *APOB* | rs12691188 | G=0,016 | G=0.061 | 3 | 0 | c.12088-46A>G | No information but deep in intron |
| 4 | *APOB* | rs1800479 | G=0,196 | G=0.142 | 28 | 4 | c.11903+50C>G | No information but deep in intron, common variant |
| 4 | *APOB* | rs139704306 | A=0,005 | A=0.002 | 1 | 0 | c.11788+16G>A | No information found |
| 4 | *APOB* | rs1801701 | T=0,098 | T=0.049 | 16 | 1 | p.(Arg3638Gln) | Associated with LDL-C in general population [8, 28], influencing BMI [30], common variant |
| 4 | *APOB* | rs533617 | C=0,027 | C=0.020 | 5 | 0 | p.(His1923Arg) | Altered binding affinity [31], probably damaging to ApoB [24], found in individuals with low TG levels [21] |
| 4 | *APOB* | rs679899 | A=0,467 | A=0.492 | 44 | 21 | p.(Ala618Val) | Probably damaging to ApoB [24] , associated with chronic kidney disease [32], associated with decreased LDL-C [8], influence on BMI [30], Ala allele associated with elevation of ApoB in hypertriglyceridemic patients [33], common variant |
| 4 | *APOB* | rs72653061 | C=0,021 | C=0.001 | 4 | 0 | c.905-15G>C | No information found |
| 4 | *APOB* | rs199702642 | T=0,005 | T=0,001 | 1 | 0 | c.694-20C>T | No information found |
| 4 | *APOB* | rs1367117 | A=0,326 | A=0.205 | 38 | 11 | p.(Thr98Ile) | Increased LDL-C [27, 8], TC levels and BMI [30, 28], predicted as benign [24], common variant [28] |
| 4 | *LDLR* | rs3745677 | A=0,112 | A=0.093 | 19 | 1 | c.190+56G>A | Possible modification of splicing efficiency negative [9], not pathogenic [11], common variant |
| 4 | *LDLR* | rs17242416 | A=0,011 | A=0.001 | 2 | 0 | c.190+144G>A | No information but deep in intron |
| 4 | *LDLR* |  | T=0,090 |  | 17 | 0 | c.817+44C>T | No information but deep in intron, common variant |
| 4 | *LDLR* | rs2738442 | T=0,060 | T=0.017 | 7 | 2 | c.1060+7T>C | No association with FH [34] |
| 4 | *LDLR* | rs12710260 | C=0,413 | C=0.283 | 32 | 22 | c.1060+10G>C | G to A substitution causes hypercholesterolemia?[9, 11], possible modification of splicing efficiency negative [9], found in Greek FH patients [35], common variant |
| 4 | *LDLR* | rs11669576 | A=0,037 | A=0.076 | 7 | 0 | p.(Ala391Thr) | Associated with increased risk for stroke [36], no effect on lipids [36], found in FH patients [35, 17, 10], polymorphism [37] |
| 4 | *LDLR* | rs1003723 | T=0,418 | T=0.282 | 33 | 22 | c.1359-30C>T | Possible modification of splicing efficiency negative [9], common variant |
| 4 | *LDLR* |  | A=0,005 |  | 1 | 0 | c.2389+23G>A | No information found |
| 4 | *LDLR* | rs2738460 | T=0,303 | T=0.250 | 33 | 12 | c.2389+46C>T | Possible modification of splicing efficiency negative [9], common variant |
| 4 | *LDLR* | rs13306501 | A=0,005 | A=0.076 | 1 | 0 | c.2389+47G>A | Possible modification of splicing efficiency negative [9] |
| 4 | *LDLR* |  | T=0,005 |  | 1 | 0 | c.*51C>T | No information, but next nucleotide exchange is considered as benign [9] |
| 4 | *LDLR* | rs14158 | A=0,282 | A=0.249 | 25 | 14 | c.*52G>A | Probably benign [9], common variant |

| **Supplementary Table 1 continuation** | | | | | | | | |
| --- | --- | --- | --- | --- | --- | --- | --- | --- |
| 4 | *LDLR* | rs3826810 | A=0,053 | A=0.073 | 10 | 0 | c.*141G>A | Probably benign [9], common variant |
| 4 | *LDLR* | rs72658873 | A=0,005 | A=0,001 | 1 | 0 | c.*281G>A | No information but far away in 3' end |
| 4 | *LDLR* | rs2738464 | G=0,114 | G=0.235 | 15 | 3 | c.*315G>C | Associated with dementia [23], common variant |
| 4 | *LDLR* | rs17249029 | A=0,005 | A=0.001 | 1 | 0 | c.*338G>A | No information but far away in 3' end |
| 4 | *LDLR* | rs2738465 | A=0,367 | A=0.322 | 31 | 19 | c.*504G>A | No information but far away in 3' end, common variant |
| 4 | *LDLR* | rs1433099 | T=0,201 | T=0.342 | 27 | 5 | c.*666T>C | No information but far away in 3' end, common variant |
| 4 | *LDLR* | rs2738466 | G=0,287 | G=0.250 | 26 | 14 | c.*773A>G | No information but far away in 3' end, common variant |
| 4 | *LDLR* |  | T=0,005 |  | 1 | 0 | c.*1022C>T | No information but far away in 3' end |
| 4 | *LDLR* | rs72658879 | A=0,043 | A=0.042 | 8 | 0 | c.*2016G>A | No information but far away in 3' end |
| 4 | *LDLR* |  | GT=0,005 |  | 1 | 0 | c.*2155_2156insGT) | No information but far away in 3' end |
| 4 | *LDLR* | rs17249078 | -=0,505 | -=0,500 | 75 | 9 | c.*2197delTA | No information but far away in 3' end, common variant |
| 4 | *LDLRAP1* | rs146180707 | C=0,005 | C=0.006 | 1 | 0 | c.245-31C>C | No information but deep in intron |
| 4 | *LDLRAP1* | rs74060930 | T=0,016 | T=0.054 | 3 | 0 | c.459+22G>T | Found in family with sever autosomal recessive hypercholesterolemia [38] |
| 4 | *LDLRAP1* | rs111288517 | A=0,022 | A=0.016 | 4 | 0 | c.459+84G>A | No information but deep in intron |
| 4 | *LDLRAP1* | rs6687605 | C=0,309 | C=0.459 | 32 | 13 | p.(Ser202Pro) | His [39] but not  Ser or Pro in this aa position cause recessive hypercholesterolemia [38, 40, 41], possibly benign, common variant |
| 4 | *LDLRAP1* | rs6688931 | A=0,446 | A=0.447 | 52 | 15 | c.616+28G>A | No information found, common variant |
| 4 | *LDLRAP1* | rs41291058 | T=0,027 | T=0.024 | 5 | 0 | p.(Arg238Trp) | Hypercholesterolaemia, autosomal recessive?[42] |
| 4 | *LDLRAP1* |  | C=0,005 |  | 1 | 0 | c.*1487T>C | No information but far away in 3' end |
| 4 | *LDLRAP1* |  | A=0,005 |  | 1 | 0 | c.*1685G>A | No information but far away in 3' end |
| 4 | *LDLRAP1* | rs7491 | T=0,413 | C=0.464 | 48 | 14 | c.*1755C>T | No information but far away in 3' end, common variant |
| 4 | *LDLRAP1* | rs4537542 | A=0,053 | A=0.021 | 10 | 0 | c.*1794G>A | No information but far away in 3' end, common variant |
| 4 | *PCSK9* | rs72658888 | A=0,011 | A=0.003 | 2 | 0 | c.-287G>A | Nucleotide exchange in next position -288G>A does not have effect on PCSK9 transcription [43] |
| 4 | *PCSK9* | rs45448095 | T=0,117 | T=0.094 | 20 | 1 | c.-64C>T | Found in hypercholesterolemic individuals [44], common variant |
| 4 | *PCSK9* | rs11583680 | T=0,106 | T=0.090 | 18 | 1 | p.(Ala53Val) | Found in FH patients [45, 46, 13, 47, 44], found in hypocholesterolemia patients [45, 47], common variant [45, 48, 49, 44] |
| 4 | *PCSK9* | rs2483205 | T=0,467 | T=0.377 | 44 | 21 | c.658-7C>T | Found in FH individuals [50, 13], common variant [44, 47] |
| 4 | *PCSK9* | rs2495477 | G=0,408 | G=0.426 | 43 | 16 | c.799+3A>G | Found in FH individuals [44, 13], common variant |

| **Supplementary Table 1 continuation** | | | | | | | | |
| --- | --- | --- | --- | --- | --- | --- | --- | --- |
| 4 | *PCSK9* | rs585131 | C=0,152 | C=0.135 | 20 | 4 | c.1355-56C>T | Common variant |
| 4 | *PCSK9* | rs562556 | G=0,147 | G=0.147 | 19 | 4 | p.(Val474Ile) | Ile474Val is associated with high TC and LDL-C [49], found in hypocholesterolemia patients [45], found in both low and high LDL-C groups [47], common variant [13, 51, 52, 45, 48, 49, 44] |
| 4 | *PCSK9* | rs505151 | A=0,936 | G=0.098 | 10 | 83 | p.(Gly670Glu) | Associated with severity of atherosclerosis [53], found in hypocholesterolemic individuals [45], associated with chronic kidney disease [32], found in normolipidemic individuals [45], found in hypercholesterolemic individuals [44], common polymorphism [48, 44, 45, 52, 47, 13], common variant |
| 4 | *PCSK9* | rs189293781 | A=0,005 | A=0.001 | 1 | 0 | c.*28G>A | No information found |
| 4 | *PCSK9* | rs28362287 | T=0,027 | T=0.024 | 5 | 0 | c.*75C>T | No information |
| 4 | *PCSK9* |  | T=0,005 |  | 1 | 0 | c.*141G>T | No information but far away in 3' end |
| 4 | *PCSK9* | rs182138201 | T=0,016 | T=0.004 | 3 | 0 | c.*234C>T | No information but far away in 3' end |
| 4 | *PCSK9* | rs17111555 | T=0,033 | T=0.024 | 4 | 1 | c.*345C>T | No information but far away in 3' end |
| 4 | *PCSK9* | rs13376071 | T=0,021 | T=0.031 | 4 | 0 | c.*414C>T | No information but far away in 3' end |
| 4 | *PCSK9* | rs72646535 | -=0,027 | -=0.024 | 5 | 0 | c.*537delT | No information but far away in 3' end |
| 4 | *PCSK9* | rs662145 | C=0,239 | C=0.272 | 34 | 5 | c.*571C>T | No information but far away in 3' end, common variant |
| 4 | *PCSK9* | rs17111557 | T=0,027 | T=0.032 | 5 | 0 | c.*614C>T | No information but far away in 3' end |
| 5 | *LDLR* | rs72658861 | C=0,005 | C=0.005 | 1 | 0 | c.1061-8T>C | Potentially hypercholesterolemia causing [54-57], substitution of T>G found in FH patients [58], unlikely to affect splicing [59], in strong LD with Thr726Ile [60, 59], also found in relatively healthy individual with normal lipid levels |
| 5 | *LDLR* | rs6413505 | T=0,005 | T=0.038 | 1 | 0 | c.1358+32C>T | Possible modification of splicing efficiency negative [9], found in relatively healthy individual with normal lipid levels |
| 5 | *LDLR* | rs72658868 | A=0,005 | A=0.009 | 1 | 0 | c.2389+41C>A | Found in relatively healthy individual with normal lipid levels |
| 5 | *PCSK9* | rs11800231 | A=0,027 | A=0.075 | 5 | 0 | c.524-11G>A | Found in FH individuals [44, 13], found in relatively healthy individual with normal lipid levels |
| 5 | *PCSK9* | rs11800243 | A=0,027 | A=0.040 | 5 | 0 | c.657+9G>A | Found in FH individuals [44, 13], found in relatively healthy individual with normal lipid levels |
|  |  |  |  |  |  |  |  |  |

| **Supplementary Table 1 continuation** | | | | | | | | |
| --- | --- | --- | --- | --- | --- | --- | --- | --- |
| 6 | *APOB* | rs676210 | A=0,181 | A=0.338 | 30 | 2 | p.(Pro2739Leu) | Hypocholesterolaemia [8, 61], associated with LDL oxidation [24], associated with VLDL-C and TG [30], common variant |
| 6 | *APOB* | rs12713844 | G=0,037 | G=0.003 | 7 | 0 | p.(Asp1113His) | Found in hypobetalipoproteinaemia individual [62], found in individuals with high and low TG levels [21] |
| 6 | *APOB* | rs12691202 | T=0,011 | T=0.022 | 2 | 0 | p.(Val730Ile) | As compound heterozygote with Arg490Trp influences severity of hypobetalipoproteinaemia [63] |
| 6 | *PCSK9* | rs11591147 | T=0,005 | T=0.009 | 1 | 0 | p.(Arg46Leu) | Associated with reduced risk to coronary heart disease [64, 52], associated with hypocholesterolemia [45, 64, 52] and increased response to statin therapy [65], found in controls [65], found in both low and high LDL-C groups [48] |

*CAT – our designated category of variant: 1 – real monogenic dyslipidemia causing mutations (described in many reports); 2 – rare (frequency in general population < 0.01), protein function damaging (based on all protein function predictors) variants; 3 – other rare (frequency in general population < 0.01), nonsynonymous and potential splice site (10 nucleotides in intron) variants; 4 – other rare (frequency in general population < 0.01), synonymous variants and common variants (frequency in general population > 0.01); 5 - variants found in relatively healthy individual with normal lipid levels; 6 – variants with opposite effect. AAF – alternative allele frequency in our cohort; FRQ – frequency of general population (*[*^http://www.ncbi.nlm.nih.gov/^*](http://www.ncbi.nlm.nih.gov/)*); HET – count of heterozygotes in our cohort; HOM – count of homozygotes in our cohort; Variant – amino acid numbering according to Human Genome Variation Society [66].*

# References
